# Activation of FXR protects against renal fibrosis via suppressing Smad3 expression

**DOI:** 10.1038/srep37234

**Published:** 2016-11-17

**Authors:** Kai Zhao, Jialin He, Yan Zhang, Zhizhen Xu, Haojun Xiong, Rujun Gong, Song Li, Shan Chen, Fengtian He

**Affiliations:** 1 Department of Biochemistry and Molecular Biology, College of Basic Medical Sciences, Third Military Medical University, Chongqing 400038, China; 2 Department of Pathogenic Biology, College of Basic Medical Sciences, Third Military Medical University, Chongqing 400038, China; 3 Division of Kidney Disease and Hypertension, Department of Medicine, Rhode Island Hospital, Brown University School of Medicine, Providence, RI 02903, USA; 4 Department of Pharmaceutical Sciences, School of Pharmacy, University of Pittsburgh, Pittsburgh, PA 15261, USA

## Abstract

Renal fibrosis is the common pathway of most chronic kidney disease progression to end-stage renal failure. The nuclear receptor FXR (farnesoid X receptor), a multiple functional transcription factor, plays an important role in protecting against fibrosis. The TGFβ-Smad signaling has a central role in kidney fibrosis. However, it remains unclear whether FXR plays direct anti-fibrotic effect in renal fibrosis via regulating TGFβ-Smad pathway. In this study, we found that the level of FXR was negatively correlated with that of Smad3 and fibronectin (a marker of fibrosis) in human fibrotic kidneys. Activation of FXR suppressed kidney fibrosis and downregulated Smad3 expression, which was markedly attenuated by FXR antagonist. Moreover, the FXR-mediated repression of fibrosis was significantly alleviated by ectopic expression of Smad3. Luciferase reporter assay revealed that FXR activation inhibited the transcriptional activity of Smad3 gene promoter. The *in vivo* experiments showed that FXR agonist protected against renal fibrosis and downregulated Smad3 expression in UUO mice. These results suggested that FXR may serve as an important negative regulator for manipulating Smad3 expression, and the FXR/Smad3 pathway may be a novel target for the treatment of renal fibrosis.

Renal fibrosis is the common pathway of most chronic kidney disease (CKD) progression to end-stage renal failure^1^,^2^,^3^. The degree of renal tubular interstitial fibrosis (RTF) is positively correlated with that of the decrease of renal function, and usually served as a significant indicator for the prognosis of CKD. Therefore, suppression of RTF should be beneficial to the treatment of renal fibrosis.

RTF is characterized by fibroblast activation, excessive production and deposition of extracellular matrix (ECM), which causes the destruction and collapse of renal parenchyma and progressive loss of kidney function. The accumulation of ECM proteins such as fibronectin (FN) is the hallmark of fibrosis, and transforming growth factor β (TGFβ) signaling has a central role in ECM production in the kidney. Moreover, it has been reported that the TGFβ1 induces ECM accumulation and tissue fibrosis via regulating Smad family members, especially Smad3^2^,^4^,^5^.

Farnesiod X receptor (FXR, NR1H4), a ligand-activated transcription factor, is highly expressed in liver, kidneys, intestine and adrenal glands^6^. Although originally being considered as a bile acid-activated transcription factor that regulates metabolism homeostasis^7^,^8^, FXR has been proposed as a novel molecular target in the treatment of inflammatory diseases^9^,^10^. Some FXR agonists have applied for the treatment of alcoholic hepatitis, nonalcoholic steatohepatitis, diabetes and primary biliary cirrhosis in clinical trials^11^,^12^,^13^,^14^. Furthermore, recent reports have indicated that FXR has anti-fibrosis function^13^,^15^,^16^,^17^,^18^,^19^. For example, FXR knockout mice display increased tendency of developing hepatic inflammation and fibrosis over time^17^,^18^. Activation of FXR has been suggested to reduce fibrosis in two experimental models of early-stage liver fibrosis either by 2 weeks bile duct ligation or 12 weeks of treatment with porcine serum^19^. Mechanistic studies show that FXR may reduce liver cell damage and fibrosis through upregulating bile salt export pump and small heterodimer partner (SHP), thereby inhibiting the production of type I collagen^19^,^20^. However, it remains unclear whether FXR can play its anti-fibrotic effect via regulating TGFβ-Smad pathway. In the present study, we demonstrated that FXR activation suppresses kidney fibrosis by downregulating Smad3 expression *in vitro* and *in vivo*, suggesting that FXR may serve as an important negative regulator for manipulating Smad3 expression, and the FXR/Smad3 pathway may be a novel target for the treatment of renal fibrosis.

## Results

### The level of FXR is negatively correlated with **that** of Smad3 and FN in human fibrotic kidney**s**

**Figure 1:**
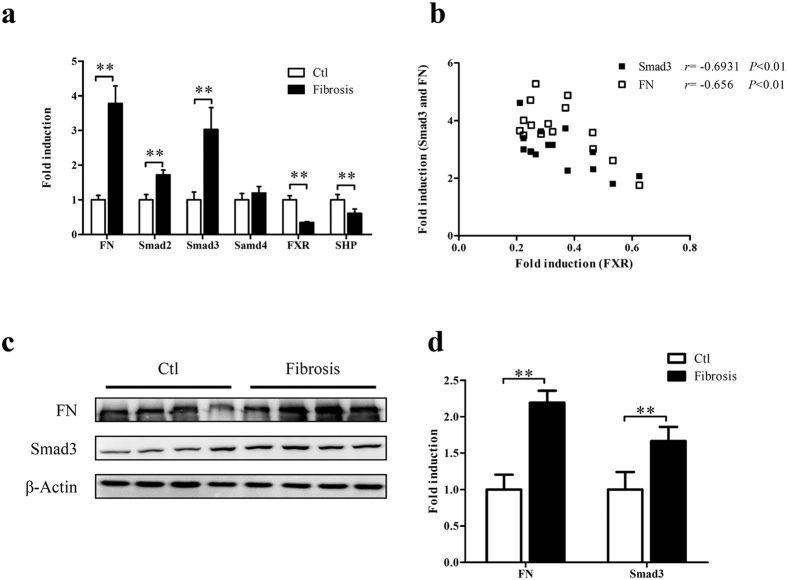
The level of FXR is negatively correlated with that of Smad3 in human fibrotic kidneys. (**a**) The expression of FXR, FN, Smad2, Smad3, Smad4 and SHP (an indicator of FXR activation) in 15 human fibrotic kidneys and 15 normal control kidney tissues (adjacent non-cancerous kidney tissues) were examined by qRT-PCR, taking β-actin as control. (**b**) The correlation between the levels of FXR and Smad3 or FN in human fibrotic kidneys were analyzed with Pearson’s test (FXR/Smad3 *r* = −0.6931, *P* < 0.01 and FXR/FN *r* = −0.656, *P* < 0.01). (**c**) The protein levels of Smad3 and FN in human fibrotic kidneys were detected by Western blot, taking β-actin as control. (**d**) Semi-quantitive analysis of the data in (**c**) with Quantity-One™ software.

To investigate the relationship between FXR and Smad3 and FN, the expression of the three molecules and SHP (an indicator of FXR activation) were examined in human fibrotic kidneys and normal control kidney tissues. As show in Fig. 1a,c,d, the fibrotic kidneys had higher levels of Smad3 and FN accompanied by the lower levels of FXR and SHP compared to the normal control kidney tissues. Correlation analysis with Pearson’s test showed that the level of FXR was negatively correlated with that of Smad3 and FN in human fibrotic kidneys (*r* = −0.6931, *P* < 0.01 and *r* = −0.656, *P* < 0.01 respectively) (Fig. 1b). Smad2 and Smad4 were also examined in these samples. The negatively correlation between FXR and Smad2 was not detected despite a higher expression of Smad2 in fibrotic tissues compared with that in normal control (Supplementary Figure S1). Smad4 had no significant change in fibrotic tissues (Fig. 1a). These results suggest that FXR may suppress Smad3 and FN and renal fibrosis.

### Activation of FXR suppresses fibrosis in human tubular epithelial cells

**Figure 2:**
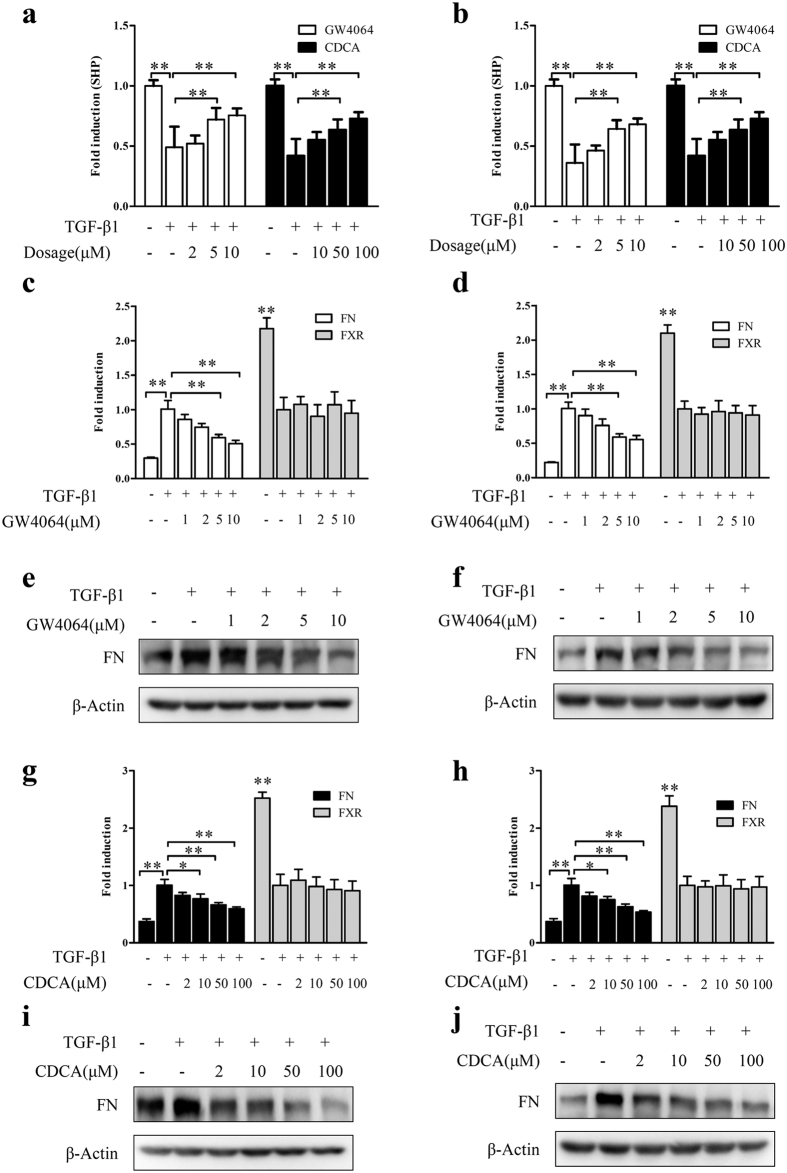
FXR activation suppresses fibrosis in human renal tubular epithelial cells. After treatment with TGF-β1 (2 μg/ml) for 24 h, HK-2 (left panel) and HKC (right panel) cells were treated with different concentrations of GW4064, CDCA or vehicle control DMSO for another 24 h. Then the mRNA levels of SHP (**a,b**), FN and FXR (**c**,**d**,**g**,**h**) were detected by qRT-PCR, and the protein level of FN (**e**,**f**,**i**,**j**) was examined by Western blot. *P < 0.05, **P < 0.01.

To investigate whether FXR activation can repress kidney fibrosis *in vitro*, human tubular epithelial cells including HK-2 and HKC cells were treated with TGFβ1 to induce cell fibrosis, and then treated with FXR agonists GW4064 and chenodeoxycholic acid (CDCA). As shown in Fig. 2a,b, both GW4064 and CDCA could upregulate SHP (but not FXR itself, Fig. 2c,d,g,h), indicating that FXR is functional in the TGFβ1-induced fibrotic cell models. Moreover, activation of FXR dramatically attenuated TGFβ1-induced FN in a dosage-dependent manner in the fibrotic cell models at mRNA level (Fig. 2c,d,g,h) and protein levels (Fig. 2e,f,i,j). These results indicated that FXR activation inhibits TGFβ1-induced fibrosis *in vitro*.

### FXR represses fibrosis in HKC cells via downregulating Smad3 expression

**Figure 3:**
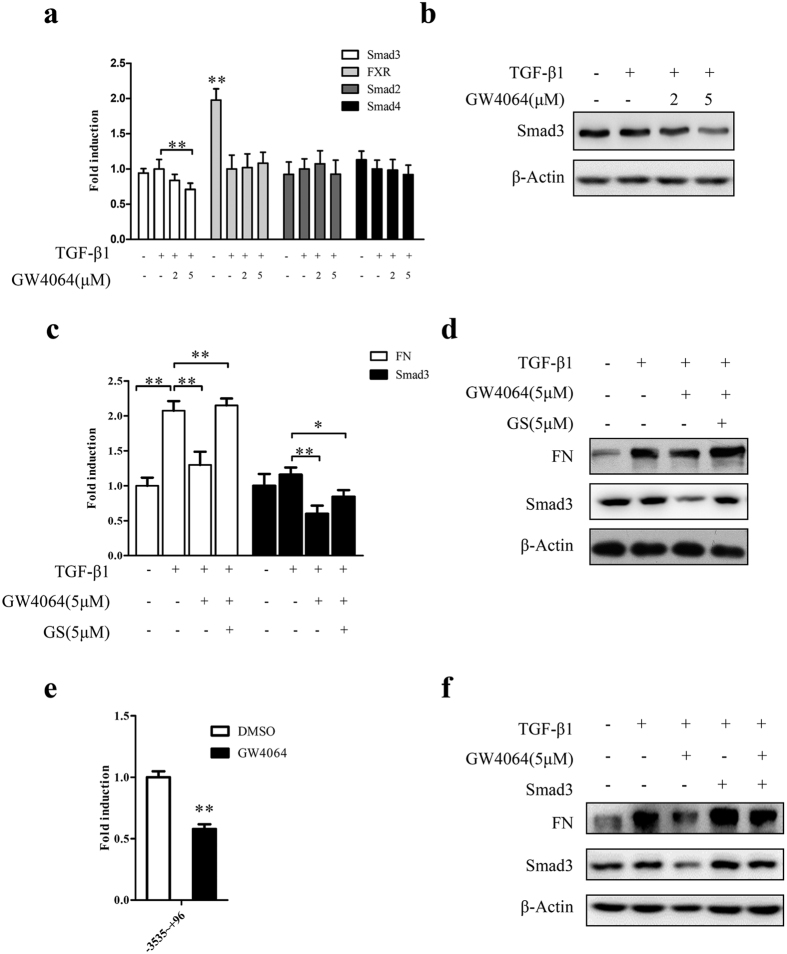
FXR represses fibrosis in HKC cells via downregulating Smad3 expression. (**a**,**b**) After treatment with TGF-β1 (2 μg/ml) for 24 h, HKC cells were treated with different concentrations of GW4064 or DMSO for another 24 h. Then the expression of Smad3, FXR, Smad2 and Smad4 were assayed by qRT-PCR (**a**) and the protein level of Smad3 was examined by Western blot (**b**). (**c**,**d**) After treatment with TGF-β1 (2 μg/ml) for 24 h, HKC cells were treated with FXR antagonist GS for 6 h. Then the cells were treated with GW4064 for another 24 h. The expression of Smad3 and FN were determined by qRT-PCR (**c**) and Western blot (**d**). (**e**) HKC cells were transiently cotransfected with the luciferase reporter pGL3-Smad3 containing Smad3 promoter region and the renilla luciferase expression vector pRL-TK for 6 h, followed by replacing transfection medium with complete medium and incubating for 12 h. Then the cells were treated with GW4064 (5 μM) or DMSO for 24 h. Subsequently, the cells were collected and luciferase activities were measured using dual-luciferase assay kit. The firefly luciferase activity was normalized with renilla luciferase activity. Data are Mean ± SD from 3 assays in triplicates. (**f**) HKC cells were transiently transfected with Smad3 expression vector pcDNA3.1-Smad3 for 6 h, followed by replacing the transfection medium with complete medium and incubating for 12 h. Then the cells were treated with TGF-β1 (2 μg/ml) for 24 h, followed by the treatment with GW4064 (5 μM) or vehicle DMSO for another 24 h. Thereafter, the protein levels of Smad3 and FN were determined by Western blot. *P < 0.05. **P < 0.01.

As shown in Fig. 3, FXR activation markedly downregulated the TGFβ1-induced Smad3 (but had no influence on the expression of FXR, Smad2 and Smad4) in HKC cells (Fig. 3a,b), which was significantly alleviated by the FXR antagonist guggulsterone (GS) (Fig. 3c,d) and the ectopic expression of Smad3 (Fig. 3f). These data indicated that the FXR-mediated inhibition of Smad3 and FN is FXR specific, and FXR represses the TGFβ1-induced cell fibrosis via decreasing Smad3 expression. Furthermore, luciferase reporter assay showed that the activation of FXR remarkably suppressed the activity of Smad3 gene promoter (Fig. 3e), indicating that FXR downregulates the expression of Smad3 at transcriptional level.

### FXR ligand protects against renal fibrosis and suppresses Smad3 expression in unilateral ureteral obstruction (UUO) mice

**Figure 4:**
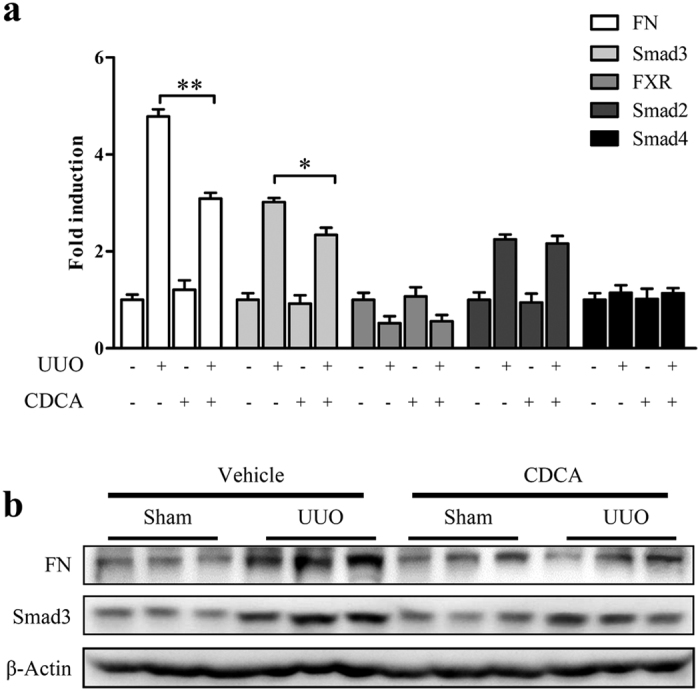
FXR ligand protects against renal fibrosis in UUO mice. The UUO and sham injury mice were intraperitoneally injected with CDCA (50 mg/kg) or vehicle control (soybean oil) (daily injection for 6 days) (5 mice in each group). At the 7th day, the mice were euthanized and the kidneys were dissected. (**a**) The mRNA levels of Smad3, FN, FXR, Smad2 and Smad4 were measured by qRT-PCR. (**b**) The protein levels of Smad3 and FN were detected by Western blot. *P < 0.05. **P < 0.01.

To evaluate the FXR-mediated inhibition of Smad3 in renal fibrosis *in vivo*, the expression of Smad3, FN and FXR was examined in the kidneys of UUO mice after intraperitoneal injection of FXR ligand CDCA. As shown in Fig. 4, treatment with CDCA dramatically decreased the levels of Smad3 and FN (Fig. 4a,b), but had no significant effect on the expression of FXR, Smad2 and Smad4 (Fig. 4a). These results indicated that activation of FXR can downregulate Smad3 and repress renal fibrosis *in vivo* in the UUO mice.

## Discussion

Renal fibrosis, particularly tubulointerstitial fibrosis, is the common end point of virtually all progressive kidney diseases. It is also a reliable predictor of prognosis and a major determinant of renal insufficiency^21^. So well understanding of the underlying mechanisms of renal fibrosis can facilitate the development of effective treatments.

Three isoforms of TGF-β have been identified in mammals, termed TGF-β1, 2 and 3, of which TGF-β1 is the most abundant isoform. Lots of evidences have shown that activation of TGF-β signaling (especially TGF-β1 signaling) plays a central role in the pathogenesis of renal fibrosis^2^,^22^,^23^,^24^,^25^. TGF-β1 promotes ECM production by activating type I and type II serine/threonine kinase receptors^26^. TGF-β1 signaling is initiated with the TGF-β receptors oligomerization and receptor-regulated Smads (R-Smads, such as Smad2 and Smad3) phosphorylation, which is followed by recruitment of common Smad (Co-Smad, Smad4) into a R-Smad/Co-Smad complex that translocates to the nucleus to regulate gene transcription^26^,^27^. Both Smad2 and Smad3 are two major downstream mediators for TGF-β1/Smad signaling in renal fibrosis^28^. However, several reports have demonstrated that knockout of Smad3 significantly alleviates kidney fibrosis, suggesting that Smad3 may be a critical pathogenic mediator of TGF-β1/Smad signaling in renal fibrosis^29^,^30^,^31^. Therefore, repression of the expression or activity of Smad3 will inhibit TGF-β1 signaling pathway, which should be helpful for the prevention and treatment of renal fibrosis. In this study, we verified that FXR activation dramatically decreases the level of Smad3, suggesting that FXR may block the signal transduction of TGF-β1 by negatively regulating Smad3 expression.

FXR, a ligand activated transcription factor and a member of the nuclear receptor superfamily in regulation of bile acid metabolism^11^,^24^, plays an important role to prevent fibrosis^25^. It has been reported that FXR activation exerts antifibrotic effects in liver by increasing the apoptosis of hepatic stellate cells^32^. Additionally, FXR prevents the progression of kidney disease in mouse models of type 1 and type 2 diabetes mellitus, and diet induced obesity and insulin resistance by regulating the metabolism of glucose and lipid^25^,^33^,^34^,^35^,^36^. However, the detailed mechanism(s) by which FXR suppresses kidney fibrosis needs to be further explored. In this study, we found that FXR inhibited renal fibrosis by downregulating Smad3 at transcriptional level. Previous studies have shown that Smad2 and Smad4 are also involved in kidney fibrosis^28^,^37^,^38^. However, in our experiments, we revealed that the expression of Smad2 and Smad4 at mRNA level no significant change after treatment with FXR ligands in the *in vitro* fibrotic cell model and *in vivo* UUO mice model, suggesting that the antifibrotic effect of FXR may not through regulating the expression of the two molecules. Of course, it needs further studies to clarify whether FXR can regulate the activity of the two molecules.

UUO is a classic model of renal interstitial fibrosis which is closely associated with high urinary tract pressure, ischemia, hypoxia, inflammatory cell infiltration and increased expression of cytokines and growth factors^39^. The UUO model of rodents can reflect the process of human obstructive nephropathy^40^. In this study, we showed that FXR ligand suppresses renal fibrosis and downregulates the expressionin of Smad3 and FN in UUO mice model. Besides the UUO renal fibrosis model, other tissue fibrosis models such as thioacetamide induced liver cirrhosis model and lithocholic acid induced lung cell fibrosis model have been reported^13^,^16^. However, it is unclear whether FXR plays its antifibrotic effects via regulating Smad3 in the other models, which needs to be further studied.

Taken together, we have revealed that there is a negatively correlation between the levels of FXR and Smad3 in kidney fibrotic patients. Moreover, FXR suppressed renal fibrosis via downregulating Smad3 *in vitro* and *in vivo*. These findings suggest that FXR may serve as an important negative regulator for manipulating Smad3 expression, and the FXR/Smad3 pathway may serve as a novel target for the treatment of renal fibrosis.

## Material and Methods

### Reagents

CDCA, GW4064 and GS were purchased from Sigma Chemical Company (St Louis, MO, USA). TGFβ1 was bought from R&D Systems, Inc. (Minneapolis, MN, USA). Lipofectamine2000, TRIzol reagent and M-MLV reverse transcriptase were from Invitrogen (Carlsbad, CA, USA). Oligo (dT) primer, Dual-luciferase assay kits, pGL3-basic and pRL-TK vectors were from Promega (Madison, WI, USA). All products for cell culture were purchased from Hycolne and Gibco (Waltham, MA, USA). Human Smad3 expression plasmid (pcDNA3.1-Smad3) was gifted from Prof. Rujun Gong (Brown University, RI, USA). Protease Inhibitor Cocktail Tablets were brought from Roche (Mannheim, Germany). PVDF Membranes and iQ^TM^ SYBR^®^ Green qPCR kits were from Bio-rad Inc. (Hercules, CA, USA). SuperSignal™ chemiluminescent substrates were purchased form Pierce Protein Biology (Waltham, MA, USA). Bradford Protein Assay Kits were from Beyotime (Beijing, China). Restriction enzymes, *Kpn* I and *Xho* I, were bought from Takara (Dalian, China).

### Clinical samples

The fibrotic kidneys and normal control kidney tissues were obtained from Department of Urinary Surgery, Daping Hospital, Third Military Medical University (Chongqing, China). The normal control kidney tissues were adjacent non-cancerous kidney tissues (at least 3 cm apart from tumor tissues). All samples were stored in liquid nitrogen immediately after dissection (see Supplementary Table S1). The fibrotic kidneys and normal control kidney tissues were identified by HE and Masson’s staining.

### Cell **c**ulture

Human proximal tubular epithelial cells HK-2 and HKC (gifted by Dr. Lorraine C. Racusen of John Hopkins University, Baltimore, MD) were maintained in Dulbecco’s modified Eagle’s medium (DMEM)/F12 containing 5% fetal bovine serum (FBS) at 37 °C in a 5% CO_2_ incubator.

### Quantitative real-time PCR (qRT-PCR)

Total RNA was extracted with TRIzol reagent and the first-strand cDNA was synthesized using SuperScript III reverse transcriptase. qRT-PCR assays for the mRNA levels of FXR, SHP, Smad2, Smad3 and FN were performed with Real-time PCR iQ^TM^ SYBR^®^ Green according to the manufacturer’s instructions, taking β-actin as an internal control. The relative expression levels of the target genes were calculated with the 2^−ΔΔCT^ method. The primer sets were listed in Supplementary Table S2.

### Western Blot

Western blot analysis was performed as described previously^41^. Briefly, the cells and tissues were lysed using RIPA buffer supplemented with protease inhibitors, and then the protein concentrations in the lysates were determined by Bradford Protein Assay Kit. Subsequently, the proteins were separated by 10% SDS-PAGE and transferred to PVDF membranes. After blocked with 5% fat-free dry milk, the membranes were incubated with primary antibodies at 4 °C overnight. Then the membranes were washed and incubated with horseradish peroxidase-conjugated secondary antibodies for 1 h. Finally, the enhanced chemiluminescence detection reagents were used to visualize the signals. The antibody against FN was purchased from Sigma (Saint Louis, MI, USA). The antibodies against Smad3 and β-actin were bought from Cell Signaling Technology (Beverly, MA, USA).

### Construction of reporter plasmid

Human *Smad*3 promoter region containing the fragment (−3535 to +96) was amplified by PCR with the oligonucleotides 5′-atcaccttcccttacagacatca-3′ (forward primer) and 5′-gcggctcccacggcgaag-3′ (reverse primer) using genomic DNA of HKC cell as template. After digestion with *Kpn* I and *Xho* I, the fragment was cloned into pGL3-basic vector and the resulting plasmid was named as pGL3-Smad3.

### Transfection and reporter assays

HKC cells with 70% to 80% confluence were transiently transfected with plasmid for 6 h using Lipofectamine 2000 in transfection medium. After replacing the transfection medium with complete medium, the cells were incubated for 12 h. Then the cells transfected with Smad3 expression plasmid pcDNA3.1-Smad3 were treated with TGF-β1 (2 μg/ml) for 24 h, followed by the treatment with GW4064 (5 μM) or vehicle control DMSO for another 24 h.

For luciferase assay, HKC cells transfected with pGL3-Smad3/pRL-TK were treated with GW4064 (5 μM) or DMSO for 24 h. Then the cells were collected and luciferase activities were examined using Dual-luciferase assay kits according to the manufacturer’s instructions. The firefly luciferase activity was normalized with renilla luciferase activity. Data are Mean ± SD from 3 assays in triplicates.

### Animal experiments with UUO mice model

Male C57BL/6 J mice aged 4–5 weeks with body weight of 20–25 g were obtained from the Laboratory Animal Center of Third Military Medical University. To establish the UUO mice model, the mice were anaesthetized by intraperitoneal injection of pentobarbital (50 mg/kg). Then the abdominal cavity was exposed via a midline incision and the left ureter was ligated at two points with 4-0 silk. The mice undergoing the same surgery except ureter ligation (sham injury) were used as control. After 24 h, the UUO and sham injury mice were intraperitoneally injected with CDCA (50 mg/kg)^42^ or vehicle control (soybean oil) (daily injection for 6 days). At the 7th day, the mice were euthanized and the kidneys were collected for qRT-PCR and Western blot analysis of the target molecules.

### Statistical **a**nalysis

All data are expressed as means ± SD unless otherwise stated. Comparisons between two groups were made with unpaired Student’s *t*-tests. Pearson’s test was used in correlation analysis between the levels of FXR and Smad3 and FN. *P* < 0.05 or *P* < 0.01 was considered statistically significant.

### Ethics statement

All animal experiments were reviewed and approved by the Animal Care and Use Committee of Third Military Medical University. All protocols used in this study were approved by the Ethics Committee of Third Military Medical University and the methods were carried out in accordance with the approved guidelines. All patients included provided written informed consent.

## Additional Information

**How to cite this article**: Zhao, K. *et al*. Activation of FXR protects against renal fibrosis via suppressing Smad3 expression. *Sci. Rep.* **6**, 37234; doi: 10.1038/srep37234 (2016).

**Publisher’s note**: Springer Nature remains neutral with regard to jurisdictional claims in published maps and institutional affiliations.

## Supplementary Material

Supplementary Information
