# Supplementary material for: Activation of FXR protects against renal fibrosis via suppressing Smad3 expression
Source: Sci Rep. 2016 Nov 17;6:37234. doi: 10.1038/srep37234 (PMC5112546; doi:10.1038/srep37234)
Supplement: Supplementary Information [file srep37234-s1.pdf]

SUPPLEMENTARY INFORMATION

Activation of FXR protects against renal fibrosis via suppressing Smad3 expression

Kai Zhao[a,b, †], Jialin He[a,†], Yan Zhang[a], Zhizhen Xu[a], Haojun Xiong[a], Rujun Gong[c], Song Li[d], Shan Chen[a,*], and Fengtian He[a,*]

[a]Department of Biochemistry and Molecular Biology, College of Basic Medical Sciences, Third Military Medical University, Chongqing 400038, China. [b]Department of Pathogenic Biology, College of Basic Medical Sciences, Third Military Medical University, Chongqing 400038, China. [c]Division of Kidney Disease and Hypertension, Department of Medicine, Rhode Island Hospital, Brown University School of Medicine, Providence, RI 02903, USA. [d]Department of Pharmaceutical Sciences, School of Pharmacy, University of Pittsburgh, Pittsburgh, PA 15261, USA. Correspondence and requests for materials should be addressed to F.H. (email: hefengtian06@aliyun.com) or S.C. (email: cs723cn@hotmail.com)

Shan Chen: Tel: +862368752258, E-mail: cs723cn@hotmail.com
Fengtian He: Tel./fax: +862368752255, E-mail: hefengtian06@aliyun.com
Department of Biochemistry and Molecular Biology, College of Basic Medical Sciences, Third Military Medical University, 30 Gaotanyan, Shapingba, Chongqing 400038, China.

Supplementary Figure S1. The correlation of FXR and Smad2 in human fibrotic kidneys. The expression of FXR and Smad2 in 15 human fibrotic kidneys and 15 normal control kidney tissues were examined by qRT-PCR, taking β-actin as a control. The correlation between the levels of FXR and Smad2 in human fibrotic kidneys were analyzed with Pearson's test (FXR/Smad4 r = -0.4332, P =0.1067).

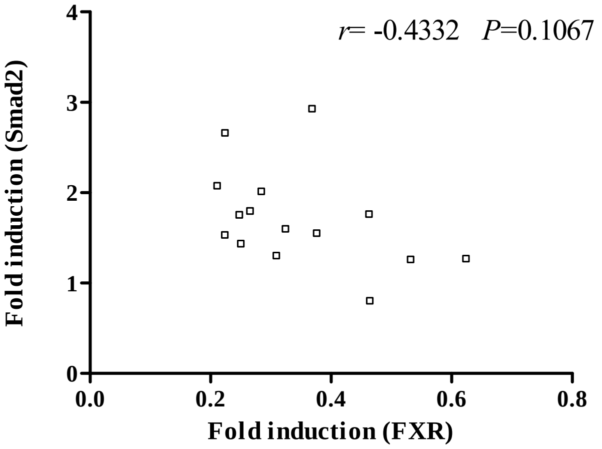

Supplementary Table S1. Subject demographics

	Control group (n=15)	Fibrosis group (n=15)
Female	3	6
Male	12	9
Average age (year)	59.6	50.3
Age(min)	35	24
Age(max)	72	80

Supplementary Table S2. The primer sets for qRT-PCR

**Gene**	**Product size (bp)**	**Forward primers**	**Reverse primers**
h*FN*	180	5′-tgtaccatcgcaaaccg-3′	5′-ggaagtcccagcagcat-3′
h*FXR*	165	5′-tacatgcgaagaaagtgtcaaga-3′	5′-actgtcttcattcacggtctgat-3′
h*SHP*	170	5′-cccaagatgctgtgaccttt-3′	5′-ccagaaggactccagacagc-3′
h*Smad2*	182	5′-cggcggagaagca-3′	5′-ttaccaaaggcagcaa-3′
h*Smad3*	120	5′-gagtgcctcagtgacagc-3′	5′-ggtagacagcctcaaagc-3′
h*Smad4*	137	5′-gaacattggatgggagg-3′	5′-taagtcaaacgcatactga-3′
h*β-actin*	157	5′-gtgaaggtgacagcagtcggtt-3′	5′-gaagtggggtggttttagga-3′
m*FN*	151	5′-gaggcaggctcagcaaat -3′	5′-tccatagcaggtacaaacc -3′
m*FXR*	148	5′-tgtgagggctgcaaaggttt-3′	5′-acatccccatctctttgcac-3′
m*Smad2*	107	5′-gaaagccatcaccactca-3′	5′-tgtcccactgatctaccg-3′
m*Smad3*	193	5′-gagaagtggtgcgagaag-3′	5′-acaggcggcagtagataa-3′
m*Smad4*	187	5′-agaacattggatggacgact-3′	5′-agacaacccgctcatagtg-3′
m*β-actin*	165	5′-tgttaccaactgggacgaca-3′	5′-ggggtgttgaaggtctcaaa-3′

Abbreviation: h, human; m, mouse.
